# Endoplasmic Reticulum Associated Aminopeptidase 2 (ERAP2) Is Released in the Secretome of Activated MDMs and Reduces *in vitro* HIV-1 Infection

**DOI:** 10.3389/fimmu.2019.01648

**Published:** 2019-07-16

**Authors:** Irma Saulle, Salomè Valentina Ibba, Enrica Torretta, Cecilia Vittori, Claudio Fenizia, Federica Piancone, Davide Minisci, Elisa Maria Lori, Daria Trabattoni, Cecilia Gelfi, Mario Clerici, Mara Biasin

**Affiliations:** ^1^Department of Biomedical and Clinical Sciences L. Sacco, University of Milan, Milan, Italy; ^2^Department of Biomedical Science for Health, University of Milan, Milan, Italy; ^3^Department of Pathophysiology and Transplantation, University of Milan, Milan, Italy; ^4^Don C. Gnocchi Foundation IRCCS, Milan, Italy; ^5^Department of Infectious Disease, ASST Fatebenefratelli Sacco, Milan, Italy; ^6^I.R.C.C.S Orthopaedic Institute Galeazzi, Milan, Italy

**Keywords:** ERAP2, haplotype, HIV-1, MDM, secretion, immune system, CTL, IFNγ

## Abstract

**Background:** Haplotype-specific alternative splicing of the endoplasmic reticulum (ER) aminopeptidase type 2 (ERAP2) gene results in either full-length (FL, haplotype A) or alternatively spliced (AS, haplotype B) mRNA. HapA/HapA homozygous (HomoA) subjects show a reduced susceptibility to HIV-1 infection, probably secondary to the modulation of the antigen processing/presenting machinery. ERAP1 was recently shown to be secreted from the plasma membrane in response to activation; we investigated whether ERAP2 can be released as well and if the secreted form of this enzyme retains its antiviral function.

**Methods:** Human monocyte derived macrophages (MDMs) were differentiated from peripheral blood mononuclear cells (PBMCs) isolated from 6 HomoA healthy controls and stimulated with IFNγ and LPS. ERAP2-FL secretion was evaluated by mass spectrometry. PBMCs (14 HomoA and 16 HomoB) and CD8-depleted PBMCs (CD8^−^PBMCs) (4 HomoA and 4 HomoB) were *in vitro* HIV-infected in the absence/presence of recombinant human ERAP2-FL (rhERAP2) protein; p24 viral antigen quantification was used to assess viral replication. IFNγ and CD69 mRNA expression, as well as the percentage of perforin-producing CD8+ T Lymphocytes, were analyzed 3 and 7-days post *in vitro* HIV-1-infection, respectively. The effect of rhERAP2 addition in cell cultures on T cell apoptosis, proliferation, activation, and maturation was evaluated as well on 24 h-stimulated PBMCs.

**Results:** ERAP2 can be secreted from human MDMs in response to IFNγ/LPS stimulation. Notably, the addition of rhERAP2 to PBMC and CD8^−^PBMC cultures resulted in the reduction of viral replication, though these differences were statistically significant only in PBMCs (*p* < 0.05 in both HomoA and HomoB). This protective effect was associated with an increase in IFNγ and CD69 mRNA expression and in the percentage of perforin-expressing CD107^+^CD8^+^ cells. RhERAP2 addition also resulted in an increase in CD8^+^ activated lymphocyte (CD25^+^HLA^−^DRII^+^) and Effector Memory/Terminally differentiated CD8^+^ T cells ratio.

**Conclusions:** This is the first report providing evidence for the release of ERAP2 in the secretome of immunocompetent cells. Data herein also indicate that exogenous ERAP2-FL exerts its protective function against HIV-1 infection, even in HomoB subjects who do not genetically produce it. Presumably, this defensive extracellular feature is only partially dependent on immune system modulation.

## Introduction

Endoplasmic reticulum aminopeptidase 2 (ERAP2) is an IFNγ- and TNFα-inducible, ubiquitously-expressed, zinc-dependent, ER-localized aminopeptidase that belongs to the M1 family of aminopeptidases. It partakes in the antigen processing pathway, hinging on the generation of peptide ligands for Major Histocompatibility Class I (MHC-I) molecules (1). In Particular, ERAP2 and the paralog ERAP1 protein are responsible for trimming the N-terminal residue extensions of the antigenic peptide precursors which are shaped in the cytosol by the proteasome, resulting in the generation of the optimal N-terminal for MHC I binding. Hence, ERAPs play a key role in editing peptide quality and, in turn, cytotoxic T lymphocyte (CTL) repertoire shaping and activation (1, 2), as indicated by studies showing reduced CTL responses in ERAP-deficient mice (3).

The haplotype network of ERAP2 is highly structured with two differentiated haplogroups, hereafter referred to as HapA and HapB. HapA leads to the expression of a 960 amino acid full-length protein (ERAP2-FL). Conversely, HapB, harbors the T allele for rs2549782 (Asn392Lys) and the G allele for rs2248374, which activates a cryptic splice site in intron 10 and the production of an alternatively spliced ERAP2 mRNA (ERAP2-AS) with two in-frame stop codons. The preservation of the two haplogroups at similar frequencies, around 0.5 in all populations, suggests a functional difference between HapA and B (4). However, the truncated version deems to be non-functional or partially functional as it exhibits a reduced aminopeptidase activity when tested *in vitro* using various aminoacyl-MCAs (5, 6). Non-synonymous ERAP2 polymorphisms were initially found to influence risk for several MHC-I-dependent diseases (ankylosing spondylitis, birdshot chorioretinopathy, and psoriasis), as well as susceptibility to different bacterial and viral infections (7–12). We showed that the G allele of rs2549782, which tags HapA, is associated with natural resistance to sexually-transmitted HIV-1 infection, potentially as a result of balancing selection through host-pathogen interactions (3, 10). Results so far obtained indicate that these variants determine a different expression of HLA-ABC on leucocytes (13, 14) and influence the processing of HIV peptides originated from intracellular processing, resulting in a unique repertoire of antigens presented to CD8+ T lymphocytes and in a diverse vulnerability to infection.

Besides its intracellular role, *in vitro* analyses demonstrated that ERAP2, like ERAP1, may be involved in extracellular biological processes, including promotion of angiogenesis and blood pressure regulation, through their role in the cleavage of bioactive peptide hormones in the renin-angiotensin system (15). The mechanism by which ERAP2 modulates these functions is puzzling given that, as far as it is known, this enzyme is only localized in the ER. Indeed, while ERAP1 secretion in the extracellular milieu has been thoroughly documented in immunocompetent cells (16, 17) and in murine macrophage cell lines (12), ERAP2 release has been reported only in the secretome of tumor cells derived from papillary thyroid carcinoma (18). The release of ERAP1 is exosome-mediated and dependent on cellular activation (19). Once secreted ERAP1 acts through a mechanism that is essentially independent from antigen processing and presentation and involves the proteolytic cutting of target proteins, which results in the activation of effector functions such as the phagocytic and NO synthetic activities of macrophages (19–21). Accordingly, Goto and coworkers described ERAP1 as a “moonlighting protein” acting as a final processing enzyme of MHC class I-presented antigenic is the case peptides in the ER, as well as a macrophage inductor in the extracellular milieu, following secretion by inflammatory stimuli.

Given that ERAP1 and ERAP2 have similar contribution to the field and a high sequence homology (22) that allows them to function both as homodimers and heterodimers (2, 23), we decided to clarify the following questions regarding ERAP2: (1) can ERAP2 be secreted by immune-competent activated cells? (2) If this is the case, does secreted ERAP2 retain its anti-HIV-1 properties? (3) Does this antiviral function rely on immune system modulation, even in the extracellular milieu?

## Materials and Methods

### PBMC Isolation and Cell Culture

Whole blood from 50 Italian Healthy Controls (HCs) was collected by venipuncture in Vacutainer tubes containing EDTA (Ethylene diamine tetracetic acid) (BD Vacutainer, San Diego, CA). Peripheral Blood Mononuclear Cells (PBMCs) were isolated by density gradient centrifugation on Ficoll (Cedarlane Laboratories Limited, Hornby, Ontario, Canada) and counted with the automated cell counter ADAM-MC (Digital Bio, NanoEnTek Inc., Korea), which allows for discrimination of viable from non-viable cells. PBMCs isolated from 8 subjects (4 HomoA and 4 HomoB) were incubated for 24-h with/without recombinant human (rh) ERAP2, in order to analyse T cell apoptosis, proliferation, activation, and maturation.

The Ethical Committee of the Don C. Gnocchi Foundation IRCCS approved the study (Prot. N°10/2018/CE_FdG/SA). All the donors signed an informed consent form, in accordance with the Declaration of Helsinki.

### Genotyping Analyses

Total DNA extracted by DNA purification Maxwell^®^ RSC Instrument (Promega) and quantified by the Nanodrop 2000 Instrument (Thermo Scientific) was used as a template for PCR amplification using TaqMan probes specifically designed to perform a SNP genotyping assay for rs2549782 (G/T) (TaqMan SNP Genotyping Assay; Applied Biosystems, Foster City, California, USA). Data were analyzed by using the allelic discrimination real-time PCR method.

### CD8^+^ T Cells Depletion by PBMCs

CD8^+^ T cells were isolated from PBMCs of 8 subjects (4 HomoA and 4 HomoB) by direct magnetic labeling using CD8 microbeads (Miltenyi Biotech, Germany) according to manufacturer's protocol. CD8^+^ isolated T cells and CD8-depleted PBMCs (CD8^−^PBMCs) were counted with the automated cell counter ADAM-MC (Digital Bio). CD8^−^PBMC purity, assessed by flow cytometry, range was always >95%. CD8^−^PBMCs were used for *in vitro* HIV-1 infection assay with rhERAP2.

### Monocyte Isolation and Monocyte-Derived Macrophages (MDMs) Differentiation

The percentage of CD14+ monocytes was determined in PBMCs isolated from 6 HomoA subjects by flow cytometer analyses. MDMs were generated as previously described (24) incubating 1 × 10^6^ adherent monocytes for 5 days in RPMI with 20% of Fetal Bovine Serum (FBS) (Euroclone) and 100 ng/ml macrophage-colony stimulating factor (M-CSF) (R&D Systems, Minneapolis, USA). MDM differentiation was assessed by optical microscope observation (Leitz Laborlux, Germany).

### Cell Cultures and MDM Protein Extraction

One million MDMs isolated from 6 HomoA subjects were cultured in RPMI (Sigma, Saint Louis, USA) with or without IFNγ (100U) (R&D Systems, Minneapolis, USA) plus LPS (1 μg/mL) (Sigma, Saint Louis, USA) and incubated for 24 h at 37°C with 5% CO_2._ At the end of the incubation period, MDMs were dislodged by non-enzymatic cell dissociation solution (SIGMA) according to manufacturer's protocol and counted with the automated cell counter ADAM-MC (Digital Bio). MDM cellular proteins were then extracted by RIPA buffer (Sigma, Saint Louis, USA) and stored at −80°C together with the collected supernatants for further analyses.

### SDS-PAGE and in-gel Tryptic Digestion

MDM extracts and MDM media protein quantification was assessed by BCA protein assay kit (Thermo Scientific Pierce). Samples were sub-pooled (50 μg per pool), separated on a 8–14%T SDS-PAGE (Hoefer) and stained with SyproRuby (Thermo Fisher). Gel bands in the range of 105–120 KDa, identified according to the HMW Calibration Kit (GE Healthcare), were gel excised and processed for in-gel tryptic digestion. Sequencing-grade modified porcine trypsin (5 ng/μL in 10 mM ammonium bicarbonate; Promega, Fitchburg, WI, USA) was used for protein digestion at 37°C, and the tryptic peptides were extracted with acetonitrile, then concentrated and dried in the vacuum concentrator.

### LC-MS/MS Analysis

Peptide samples were reconstituted in HPLC buffer A (0.1% formic acid) and separated on a Dionex UltiMate 3000 HPLC System with an Easy Spray PepMap RSLC C18 column (150 mm, internal diameter of 75 μm) (Thermo Scientific), adopting a four steps ACN/formic acid gradient (8% ACN in 0.1% formic acid for 5 min, 8–40% ACN in 0.1% formic acid for 90 min, 40–90% ACN in 0.1% formic for 1 min, 90% ACN for 15 min, at a flow rate of 0.3 μl/min), and electrosprayed into an Orbitrap Tribrid Fusion (Thermo Fisher Scientific, Bremen, Germany). The LTQ-Orbitrap was operated in positive mode in data-dependent acquisition mode to automatically alternate between a full scan (350–2,000 m/z) in the Orbitrap (at resolution 60000, AGC target 1000000) and subsequent CID MS/MS in the linear ion trap of the 20 most intense peaks from full scan (normalized collision energy of 35%, 10 ms activation). Isolation window: 3 Da, unassigned charge states: rejected, charge state 1: rejected, charge states 2+, 3+, 4+: not rejected; dynamic exclusion enabled (60 s, exclusion list size: 200). Mass spectra were analyzed using MaxQuant software (version 1.6.3.3). The initial maximum allowed mass deviation was set to 6 ppm for monoisotopic precursor ions and 0.5 Da for MS/MS peaks. Enzyme specificity was set to trypsin/P, and a maximum of two missed cleavages were allowed. Carbamidomethylation was set as a fixed modification, while N-terminal acetylation and methionine oxidation were set as variable modifications. The spectra were searched by the Andromeda search engine against the Homo Sapiens Uniprot sequence database (release 22.10.2018). Protein identification required at least one unique or razor peptide per protein group. Quantification in MaxQuant was performed using the built in XIC-based label free quantification (LFQ) algorithm using fast LFQ. The required false positive rate (FDR) was set to 1% at the peptide, 1% at the protein and 1% at the site-modification level, and the minimum required peptide length was set to 7 amino acids.

### *In vitro* HIV-1 Infection Assay of Recombinant Human (rh) ERAP2-Treated PBMCs and CD8^−^PBMCs

Recombinant human (rh)-ERAP2 (R&D Systems, Minneapolis, USA) resembles the full length ERAP2 variant and preserves the same enzymatic activity *in vitro* (25). To assess the optimal dose of rh-ERAP2 to be used in an *in vitro* HIV-1 infection assay, 2 × 10^6^ PBMCs isolated from 3 GG (HapA) HCs were cultured in RPMI 1640 containing FBS (20%) with or without different doses (10, 100, 1000 ng/ml) of rh-ERAP2 (R&D Systems, Minneapolis, USA). After 3 h, 0.5 ng HIV-1_Ba−L_ virus /1 × 10^6^ cells were added to each well and incubated for 24 h at 37°C and 5% CO_2_. Cells were then washed and re-suspended in medium containing IL-2 (15 ng/ml) (R&D systems, Minneapolis, Minnesota, USA), RPMI 1640, 20% FBS with/without different doses of rhERAP2 and incubated at 37°C and 5% CO_2_. Every 2 days, cells were supplemented with IL-2 and rhERAP2. p24 antigen ELISA (XpressBio, Frederick, MD, USA) was performed on 6-day post infection supernatants. After assessing the optimal dose to be used (100 ng/ml), the same assay was performed on PBMCs (14 HapA and 16 HapB) and CD8^−^PBMCs (4 HapA and 4 HapB). Furthermore, PBMCs were collected 3 and 6 days post infection for mRNA expression and cytometric analyses, respectively.

### Gene Expression Analysis

RNA analyses were made as previously described (26). cDNA quantification for CD69 and IFNγ, (Bio-rad, CA, USA) was performed by using a real-time PCR (CFX96 connect, Bio-rad, CA, USA) and a SYBR Green PCR mix (Bio Rad), and all reactions were run in duplicate. The results are presented as the media of the relative expression units to the glyceraldehyde-3-phosphate dehydrogenase (GAPDH) and β-actin reference genes calculated by the 2^−ΔΔ*Ct*^ equation using the CFX manager 3.1 (Bio Rad). Reactions were performed according to the following thermal profile: initial denaturation (95°C, 15 min) followed by 40 cycles of 15 s at 95°C (denaturation) and 20 s at 60°C (annealing) and 20 s at 72°C (extension). Melting curve analysis was also analyzed for amplicon identification. Ct values of 35 or higher were excluded from the analyses.

### Immunofluorescent Staining

PBMCs were stained with Phycoerythrin-Cyanin-5 (PC5)-labeled anti-CD4, Phycoerythrin-Cyanin-7 (PC7)-labeled anti-CD8, PC7-labeled anti-CD14, PC5-labeled anti-CD25-, CD45RA-, CD107A, Phycoerythrin (PE)-labeled anti-CCR7, PE-labeled anti-HLA-DRII and Fluorescein Isothiocyanate (FITC)-labeled anti-Annexin V specific mAbs for 30 min at RT in the dark, washed with PBS and fixed with 100μl of PFA (1%) (BDH, UK). For the analysis of intracellular protein expressing cells, PBMCs were washed, treated with Cell Permeabilization kit (FIX & PERM kit, eBioscience) and incubated for 30 min at 4°C in the dark with allophicocianine (APC)-labeled anti-Perforin specific monoclonal antibodies.

### Evaluation of Apoptosis

CD4-PC5 and CD8-PC7 stained cells were washed and resuspended in ice-cold 1X Binding Buffer (Beckman-Coulter, Fullerton, CA, USA) plus 10μl of Annexin V (AV) for 15 minutes on ice in the dark. Finally, cell suspensions were resuspended in 400ul of 1X Binding Buffer and analyzed by flow-cytometry.

### Carboxyfluorescein Diacetate Succinimidyl Ester (CFSE) Proliferation

Cells were either left unstained as a control or stained with CFSE (Cell-Trace CFSE Proliferation Kit, Molecular Probes, Invitrogen technologies) to a final 10 μM concentration with PBS, and immediately incubated for 3 min. Cells were then washed and incubated RPMI-1640 culture media with 20% of human serum and adjusted to a concentration of 1 million per ml. Cells were then cultured unstimulated or were stimulated with 100ng/mL of rhERAP2. After 5 days, the cells were harvested and the CFSE signal of gated lymphocytes was analyzed by flow cytometry.

### Cytometric Analysis

Analyses were performed using a Beckman-Coulter Gallios flow-cytometer equipped with two lasers operating at 488 and 638 nm, respectively, interfaced with Gallios software and analyzed with Kaluza v 1.2. Two-hundred thousand events were acquired and gated on Forward and Side scatter properties for lymphocyte. Data were collected using linear amplifiers for forward and side scatter and logarithmic amplifiers for FL1, FL2, FL4, FL5, and FL6. Samples were first run using isotype control or single fluorochrome-stained preparations for color compensation. Rainbow Calibration Particles (Spherotec, Inc. Lake Forest, IL) were used to standardize results.

### Statistical Analyses

Data were analyzed using Student's T or ANOVA test by GRAPHPAD PRISM version 5 (Graphpad software, La Jolla, Ca, USA), and *p*-values of 0.05 or less were considered to be significant.

## Results

### ERAP2 Allelic Variants

Analysis of ERAP2 SNP prevalence in the selected genes did not show any difference compared with the European population distribution reported in the U.S. National Library of Medicine Database [https://www.ncbi.nlm.nih.gov/snp/rs2549782?fbclid=IwAR1ZdwC747PDWvtAzt6hZBV5j7oFZiPkLjY-JdSee1Plzvym7fhJVQc1Aks (Data not shown). Among the 50 genotyped subjects: 14 were HomoA, 20 heterozygous, 16 HomoB. Successive analyses were performed only on PBMCs isolated from HomoA and HomoB subjects to avoid confounding results, possibly associated to the heterozygous genotype.

### ERAP2 Is Secreted by LPS/IFNγ Stimulated Human MDMs

As ERAP1 is released in the secretome of immunocompetent cells following IFNγ and LPS stimulation, we hypothesized that its paralog ERAP2 might also be secreted into the extracellular milieu of activated MDMs. To this end, high resolution MS analysis was conducted on both MDM supernatants and cellular proteins after one-dimensional SDS PAGE, on gel bands close to ERAP1 and ERAP2 molecular weight (107 and 110 KDa).

**Figure 1:**
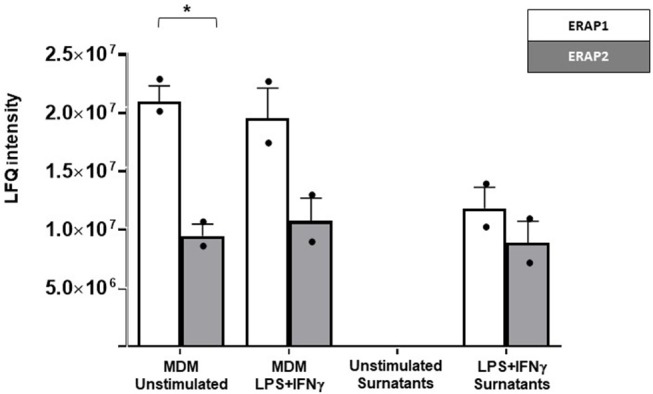
Label free quantitation (LFQ) intensity of ERAP2 and ERAP1 in unstimulated and LPS/IFN-γ stimulated MDMs from HomoA subjects and in related cell media. ERAP1 (white bars) was generally more expressed than ERAP2 (gray bars), with a statistical *p*-value of 0.02 in unstimulated cells. ERAP1 and ERAP2 were not secreted by unstimulated MDMs but only in response to macrophage activation by LPS/ IFNγ. Mean values and SEM are shown. ^*^p < 0.05.

ERAP1 and ERAP2 expression was detected in both unstimulated and stimulated MDM cellular extracts (Figure 1; Figure S1; Table S1). Notably, co-stimulation of human MDMs with IFNγ and LPS induced ERAP1 as well as ERAP2-secretion. As expected, ERAP1 expression was higher compared to ERAP2 in all the conditions (27), and these differences reached statistical significance in unstimulated cellular extracts (*p* < 0.02).

To exclude the possibility that ERAPs release was due to an aspecific mechanism resulting from cell damage, the presence of ERAP2 and ERAP1 proteins was also investigated in cell media of unstimulated MDMs. Both proteins were totally absent in the secretome of unstimulated MDMs (Figure 1). These results show for the first time that ERAP2 is secreted by cells and that its secretion is a consequence of macrophage activation.

### rhERAP2 Addition Reduces *in vitro* HIV-1 Infection in a Dose Dependent Manner in PBMCs

We next evaluated whether rhERAP2 pre-treated PBMCs could modulate viral susceptibility/replication in an *in vitro* HIV-1 infection assay.

**Figure 2:**
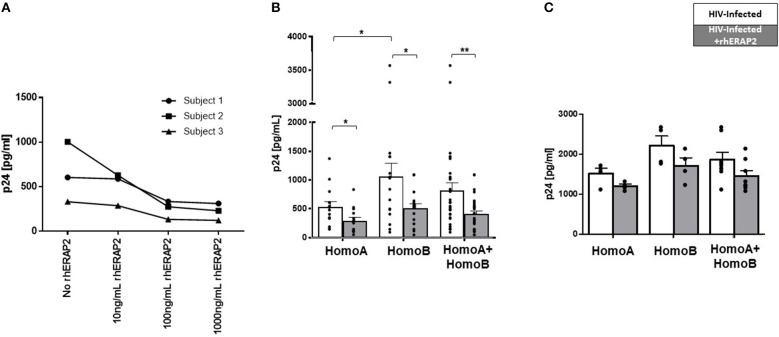
Susceptibility to *in vitro* HIV-1 infection was reduced in peripheral blood mononuclear cells (PBMCs) and CD8-depleted PBMCs (CD8^−^PBMCs) treated with recombinant human ERAP2 (rhERAP2). **(A)** PBMCs from 3 subjects were treated with different doses of rhERAP2 (10, 100, 1,000 ng/ml) and *in vitro* infected with a R5 HIV1Ba-L. **(B)** PBMCs from 14 HomoA and 16 HomoB individuals were untreated (white bars) or treated (gray bars) with 100 ng/ml of rhERAP2 and *in vitro* infected with a R5 HIV1Ba-L. **(C)** CD8^−^PBMCs from 4 HomoA and 4 HomoB individuals were untreated (white bars) or treated (gray bars) with 100 ng/ml of rhERAP2 and *in vitro* infected with a R5 HIV1Ba-L. P24 concentration was measured by ELISA 6 days post *in vitro* HIV-1 infection supernatants in all the experiments. Mean values and SEM are shown. ^*^p < 0.05; ^**^p < 0.01.

Initial results obtained in a pilot study performed on PBMCs from 3 HomoA subjects, demonstrated that rhERAP2 reduces HIV-1 replication in a dose-dependent manner with a peak effect using 100 ng/ml of the enzyme (Figure 2A) (*p* < 0.05).

Subsequently, PBMCs from 14 HomoA and 16 HomoB subjects were HIV-1 infected *in vitro* in the absence/presence of 100 ng/ml of rhERAP2. Six days after *in vitro* infection, and confirming previously published results (28), p24 levels were significantly lower in HomoA compared to HomoB PBMC cultures in which rhERAP2 was not added to the culture medium (*p* < 0.05). Remarkably, the addition of rhERAP2 drastically reduced viral replication in HomoB cells (percentage of reduction compared to cells cultured without rhERAP2: 48%; *p* < 0.03), which do not genetically produce the full-length form of the protein (Figure 2B). rhERAP2 could also further down-regulate susceptibility to HIV-1 infection/replication in HomoA cells (percentage of reduction compared to cells cultured without ERAP2: 45%; *p* < 0.03) (Figure 2B), i.e., cells that naturally produced the full-length form of the protein. These results indicate that the antiviral properties of full-length ERAP2 are additive and can be modulated.

Considering all the analyzed subjects independently of their ERAP2 genotype, the protective effect exerted by rhERAP2 (>2-fold p24 decrease) was observed in 21 out of 30 individuals (Figure 2B). Thus, ERAP2-FL preserves its antiviral function even when secreted in the extracellular milieu.

Finally, we further investigated CD8^+^ T cell role in rhERAP2-treated PBMCs by replicating the *in vitro* HIV-1 infection assay on CD8^−^PBMCs isolated from 8 subjects (4 HomoA and 4 HomoB) in the presence/absence of rhERAP2. Results showed that even in the absence of CD8^+^ T cells, rhERAP2 addition to cell cultures resulted in a partial reduction of p24 levels in both HomoA and HomoB subjects (percentage of reduction compared to cells cultured without rhERAP2: HomoA 21%, HomoB 23%), however, these differences did not reach statistical significance (Figure 2C). Such results suggest that the antiviral effect mediated by rhERAP2 is partially dependent on CD8^+^ cell activation but it also relies on the activation of other effector mechanisms, which are maintained in CD8^−^PBMCs.

### rhERAP2 Antiviral Activity Is Only Partially Dependent on Cytotoxic Response

The antiviral activity of intracellular ERAP2-FL protein presumably involves its ability to shape a peptide repertoire that activates a quantitatively and/or qualitatively more advantageous pool of CD8^+^ T cells. To verify whether even exogenous ERAP2 modulates CTL response, we analyzed IFNγ and CD69 mRNA expression, which are routinely monitored as CTL's effector and activation markers, respectively.

**Figure 3:**
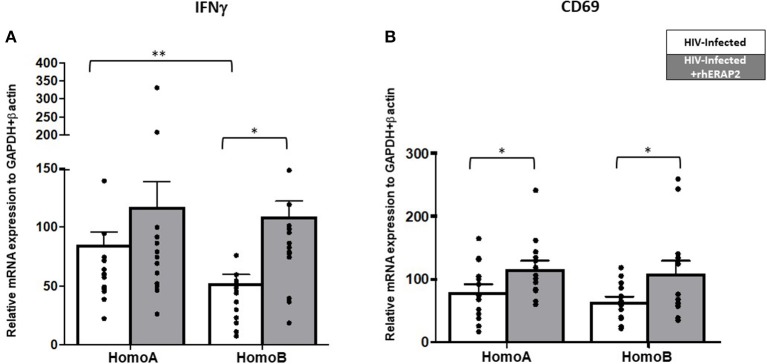
mRNA expression of interferon-γ (IFNγ) and CD69 was increased in *in vitro* rhERAP2-treated HIV-1 infected PBMCs. IFNγ **(A)** or CD69 **(B)** mRNA expression in rhERAP2 untreated (white bars) or treated (gray bars) PBMCs from HomoA and HomoB subjects 3 days after *in vitro* HIV-1 infection. Mean values and SEM are shown. ^*^p < 0.05; ^**^p <0.01.

IFNγ mRNA expression was significantly higher in HomoA HCs independently of rhERAP2 addition (*p* < 0.03) (Figure 3A). Exogenous addition of rhERAP2 to cell cultures resulted in an upregulation of IFNγ mRNA expression in *in vitro* HIV-1 infected PBMCs of all the subjects enrolled in the study; this increase was statistical significant in HomoB cells alone (*p* < 0.001) (Figure 3A).

CD69 expression level was comparable in rhERAP2-untreated PBMCs of HomoA and HomoB individuals; exogenous addition of rhERAP2 to cell cultures significantly increased CD69 expression in both HomoA and HomoB cells (*p* < 0.05 in both cases) (Figure 3B).

**Figure 4:**
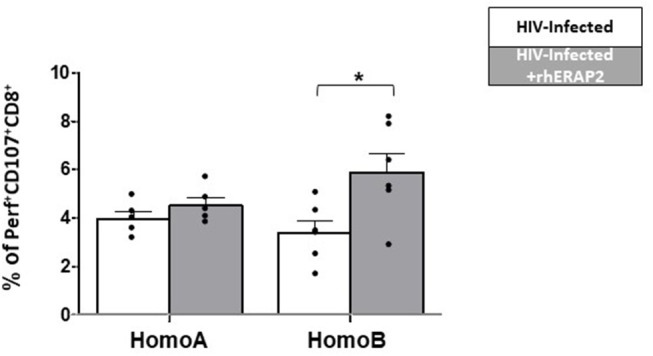
The percentage of perforin-expressing CD107^+^CD8^+^ cells was increased in *in vitro* rhERAP2-treated HIV-1 infected PBMCs. Percentage of perforin-expressing CD107+CD8+ cells in rhERAP2 untreated (white bars) or treated (gray bars) PBMCs from HomoA and HomoB subjects 3 days after *in vitro* HIV-1 infection. Mean values and SEM are shown. ^*^p < 0.05.

ERAP1 treatment significantly increased levels of cytotoxic cell activation, as evidenced by increased CD107α expression (29). Notably, in our study, perforin-producing CD107^+^CD8^+^ cells in untreated cultures were slightly increased in HomoA compared to HomoB cells, although these differences did not reach statistical significance (Figure 4). As shown in Figure 4, in HomoA subjects, no significant differences were observed in CD107^+^CD8^+^ cytotoxic T lymphocytes after exogenous addition of rhERAP2 to cell cultures. Conversely, in HomoB individuals, the addition of rhERAP2 to cell cultures resulted in a significant increase of perforin-producing CD107^+^CD8^+^ cells.

These results indicate that the antiviral activity of extracellular ERAP2-FL protein is partially dependent on activation of cell-mediated immune response, mainly in those subjects who do not genetically produce a functional ERAP2 protein.

### rhERAP2 Effect on T Cell Apoptosis, Proliferation, Activation, and Maturation

Since the antiviral activity of extracellular rhERAP2-FL protein seems to be partially dependent on activation of cell-mediated immune response, we next investigated the effect exerted by rhERAP2 on T cell apoptosis, proliferation, activation and maturation following PBMC incubation with/without rhERAP2 for 24 h. Analyses were performed on 4 HomoA and 4 HomoB subjects.

#### T Cell Apoptosis

rhERAP2 did not induce apoptosis either in CD4+or in CD8+ T cells as CD4+AV+ and CD8+AV+ cells were comparable in untreated and rhERAP2-treated conditions. Moreover, even after dividing the enrolled subjects according to their genotype, we did not observe any effect on apoptosis induction (Figure S2).

#### T Cell Proliferation

No differences were observed in untreated and rhERAP2-treated CD4^+^ and CD8^+^ T cell proliferation. This result suggests that the antiviral mechanism exerted by rhERAP2 does not depend on the modulation of possible target cell's replication (Figure S3).

#### T Cell Activation

**Figure 5:**
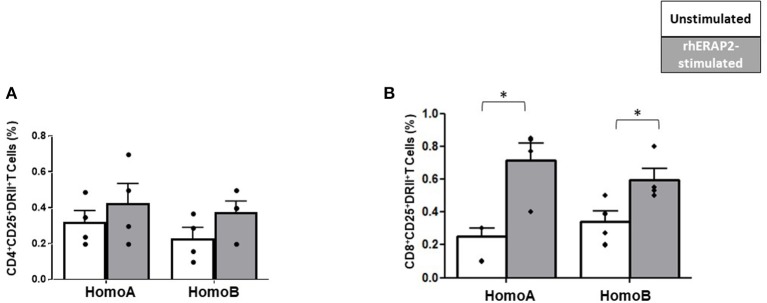
The percentage of CD25^+^HLA-DRII^+^CD8^+^ was increased in *in vitro* rhERAP2-treated PBMCs. CD25^+^HLA-DRII^+^CD4^+^ T cells **(A)** or CD25^+^HLA-DRII^+^CD8^+^ T cells **(B)** in 24 h-rhERAP2 untreated (white bars) or treated (gray bars) PBMCs from 4 HomoA and 4 HomoB subjects. Mean values and SEM are shown. ^*^p < 0.05.

Following rhERAP2 stimulation, the percentage of CD25^+^HLA-DRII^+^CD4^+^ T cells was slightly increased in both HomoA and HomoB cells, although not significantly (Figure 5A). Conversely, the percentage of CD25^+^HLA-DRII^+^CD8^+^ T cells was significantly increased in both Homo A (*p* < 0.01) and HomoB (*p* < 0.03) subjects (Figure 5B).

#### T Cell Maturation

**Figure 6:**
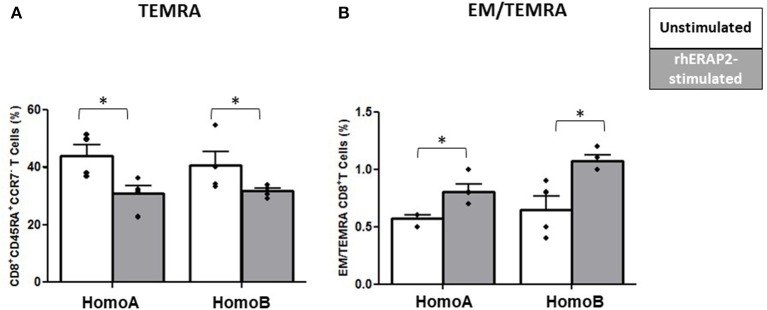
The percentage of CD8^+^ effector memory re-expressing CD45RA (TEMRA: CD45RA^+^CCR7^−^) T cells was reduced in *in vitro* rhERAP2-treated PBMCs. **(A)** Percentage of CD8+ TEMRA in 24 h-rhERAP2 untreated (white bars) or treated (gray bars) PBMCs from 4 HomoA and 4 HomoB subjects. **(B)** Ratio between EM (Effector Memory CD45RA^−^CCR7^−^) and TEMRA CD8+ T cells in 24 h-rhERAP2 untreated (white bars) or treated (gray bars) PBMCs from 4 HomoA and 4 HomoB subjects. Mean values and SEM are shown. ^*^p < 0.05.

No differences were observed in the percentage of CD4^+^ Naïve (TN: CD45RA^+^CCR7^+^), CD4^+^ Central Memory (CM: CD45RA^−^CCR7^+^), CD4^+^ Effector Memory (EM: CD45RA^−^CCR7^−^) and CD4^+^ effector memory re-expressing CD45RA (TEMRA: CD45RA^+^CCR7^−^) T cells. Conversely, CD8^+^ T cell maturation was affected by rhERAP2 treatment. In particular, the percentage of TEMRA CD8+ T cells was significantly reduced in both HomoA (p <0.03) and HomoB subjects (*p* < 0.05) following rhERAP2 treatment (Figure 6A). As a consequence, the ratio between EM and TEMRA CD8^+^ T cells was significantly increased in rhERAP2 treated PBMCs (Figure 6B).

## Discussion

There is a strong body of evidences highlighting that ERAP1 and ERAP2 play a fundamental role in precursor peptide proteolysis within the ER, which represents their natural domicile. However, as these aminopeptidases are involved in other biological processes, such as shedding of cytokine receptors, post-natal angiogenesis, and regulation of blood pressure (9), the overall subcellular localization of ERAPs remains debatable (15, 29). Recent results indicated that ERAP1 can be localized in the cytosol (30), at the cell membrane as a type II integral membrane protein (31), and can be released from the cell following appropriate activation (20, 32). Therefore, ERAP1 exhibits an outstanding adaptability in terms of subcellular localization, which is mainly conditioned by the cell type expressing it and by the environmental stimuli triggering the cell (i.e., stress response). As for ERAP2, a multidisciplinary integrated analysis revealed its overexpression, along with other candidate genes, in papillary thyroid carcinoma (PTC) and the cystic fluid counterpart, pointing out a possible translocation from the canonical ER localization (18). However, as tumor cells are by definition genetically unstable and the cancer secretome may significantly differ from that of the physiological counterpart (32), to our knowledge this is the first report demonstrating ERAP2 secretion by immunocompetent cells.

Notably, ERAP2, likewise ERAP1 could be detected only in media of MDMs triggered by inflammatory stimuli, indicating that the secretion of these proteins is a tightly controlled process which is activated exclusively in response to a specific input. The molecular mechanism leading to ERAP2 secretion *in vivo* will need to undergo specific analyses. Previous studies focusing on ERAP1 nevertheless allow some speculations. Thus, ERAP1 does not possess any classical ER localization signal and its retention in the ER depends on the interaction with Erp44, an ER resident protein disulfide isomerase, through the formation of a mixed disulfide bond with a cysteine residue in the exon 10 loop (33). This interaction was shown to be important for regulating ERAP1 retention and secretion, and in that context, in controlling blood pressure through angiotensin II cleavage (33). As ERAP2 lacks an ER retention signal as well and its exon 10 has a crystallography structure similar to that of ERAP1 (34), its secretion might be promoted similarly to what is described for ERAP1. However, to our knowledge, an interaction between ERAP2 and ERp44 has not yet been demonstrated and further analyses are needed to disclose the molecule/s holding ERAP2 in the ER. Furthermore, in line with ERAP1, it is also possible to assume that two signals are required for the secretion of ERAP2. The first one, represented by IFNγ, is responsible for the synthesis and accumulation of the aminopeptidases within the ER but not for their release, as it probably modifies the amount and/or composition of the ER retention machinery of ERAPs. The second one, provided by LPS, delivers the stress signal indispensable to induce ERAP secretion and possibly their extracellular activation.

In the attempt to investigate the biological functions of ERAP2 in the secretome, we first verified if its anti-HIV activity, recently associated to the intracellular counterpart (28), is preserved even in the extracellular milieu. Notably, results showed that the addition of rhERAP2 to the *in vitro* HIV-1 infection assay significantly reduced HIV-1 replication not only in HomoA, but also in HomoB cells, that do not genetically produce it. The mechanism by which exogenous ERAP2 interferes with HIV-1 infection/replication is under investigation. However, the analyses so far performed suggest that addition of rhERAP2 to PBMCs did not affect CD4^+^ and CD8^+^ T cell proliferation and apoptosis but, conversely, resulted in an increased activation of T cells, mainly CD8^+^CD25^+^DRII^+^, as well as in a decreased percentage of TEMRA CD8^+^ T cells in both HomoA and HomoB subjects. As a consequence, the ratio between EM and TEMRA CD8+ T cells was significantly increased in rhERAP2 treated PBMCs, suggesting a reduced differentiation of central memory CD8+ T cells into TEMRA in favor of EM. Although the molecular mechanism(s) responsible for the effect of rhERAP2 on CD8^+^ T cell maturation need to be further investigated, we can speculate on the antiviral effect resulting from this altered CD8+ T cell maturation. Thus, both EM and TEMRA cells strongly express genes involved in CD8+ T cell effector function, but EM cells have a greater expansion potential, as well as increased IL-2 and IFNγ production, than TEMRA ones (35, 36). Moreover, while TEMRA are short lived cells that can no longer differentiate further to adopt another identity, EM cells can revert to long-lived memory cells, which are key for protecting the individual from severe reinfection (37). Furthermore, mRNA expression of the T cell activation marker CD69, the effector molecule IFNγ and the percentage of perforin-expressing CD8^+^ T cells significantly increased following rhERAP2 addition to *in vitro* HIV-1 infected PBMCs. As mitogen-activated protein (MAP) kinases have been implicated in many CD8+ T cell physiological processes, including cell proliferation, differentiation, and death (38) and p38 MAP kinase regulates IFNγ production in CD8+ T cells (39), the investigation of T cell MAP kinase signalosome is necessary to further clarify the ERAP2-HIV-infection axis. Interestingly, this feature was definitely more evident in subjects who are not able to genetically synthetize ERAP2 (HomoB subjects), presumably following rhERAP2 internalization and re-localization into the ER, where it compensates for the natural absence of the protein. Conversely, rhERAP2-mediated inhibition of *in vitro* HIV-1 infection was clearly more potent in HomoA cells, in whom rhERAP2 administration resulted in a moderate CTL activation. Notably, HIV replication was reduced by rhERAP2 addition even in CD8^+^ T cells- depleted cultural conditions. These results suggest that, even if CD8+ T cells play a pivotal role in mediating the antiviral effect of ERAP2, such an effect also relies on the activation of other effector mechanisms, which are maintained in CD8^−^PBMCs.

**Figure 7:**
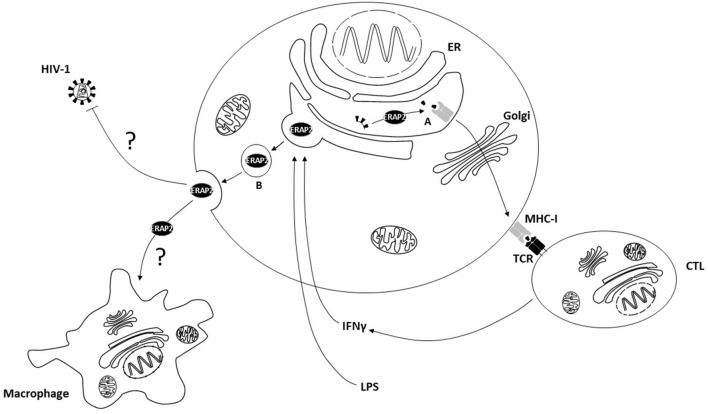
The cellular localization and functions of ERAP2. **(A)** Intracellular ERAP2 plays a key role in editing peptide quality and, in turn, cytotoxic T lymphocyte (CTL) repertoire shaping and activation. **(B)** Following interferonγ (IFNγ) and lipopolysaccharides (LPS) stimulation ERAP2 may be released in the secretome of immune competent cells where it interferes with HIV-1 replication through direct or indirect mechanisms, possibly involving macrophage activation. ER, Endoplasmic Reticulum; MHC-I, Major Histocompatibility Complex class I; TCR, T Cell Receptor.

As a whole, these data suggest a scenery in which exogenous ERAP2 antiviral function relies on two independent but addictive mechanisms (Figure 7). Thus, extracellular ERAP2 may be partially endocytosed by the cells, where it displays its canonical function in immune system modulation. This protein, however, can also interact with extracellular substrates resulting in the stimulation of antiviral pathways that are only partially identified (6). In this context, some information may derive from previous data obtained by analyzing ERAP1. Thus, ERAP1, once secreted by different cell lines (i.e., COS-7, RAW264.7 cells), is able to trim unpredictable molecules, contributing to the activation of a signaling cascade but not necessarily associated with the immune system. Hattori et al., for example, proved an efficient ERAP1 cleavage activity of angiotensin II to angiotensin III and IV (16). “Bystander cells” activation could also contribute to mediate recERAP2 antiviral function. Indeed, secreted ERAP1 was shown to enhance macrophage phagocytosis (20) and nitric oxide (NO) synthesis by trimming peptides with N-terminal arginine (Arg) residues (21). Since ERAP2 shows a strong preference for basic arginine residue trimming (2) and supplies of free-Arg are essential for maximum NO synthesis (21), it is tempting to speculate that stressor-induced ERAP2 secretion could have direct and indirect effects on macrophage activation, or more broadly on pro-inflammatory response. This, in turn, could constitute a favorable environment, raising an immune barrier that could interfere with HIV entry, as suggested by several studies portraying the profile of subjects who naturally resist HIV-1 infection (40–46).

Open questions concerning, among others, ERAP1 and ERAP2 dimerization in the secretome, as well as the identification of the substrates recognized by ERAP2 within the ER and in the extracellular milieu, require additional researches. However, the putative relevance of ERAP2 secretion should not be underestimated. This finding poses the premises to further investigate the role of ERAP2 in both innate and adaptive immunostimulatory pathways, and suggests that ERAP2 can promote inflammation via alternative mechanisms in addition to its well-characterized antigen processing function.

## Data Availability

The raw data supporting the conclusions of this manuscript will be made available by the authors, without undue reservation, to any qualified researcher.

## Ethics Statement

The Ethical Committee of the Don C. Gnocchi Foundation IRCCS approved the study (Prot. N°10/2018/CE_FdG/SA). All the donors signed an informed consent form, in accordance with the Declaration of Helsinki.

## Author Contributions

MB and MC conceived the study. IS and MB wrote the paper. IS, SI, EL, ET, CV, FP, and DT performed the experiments and analyzed the data. DM enrolled and took blood samples from healthy controls. CF and CG critically revised the paper.

### Conflict of Interest Statement

The authors declare that the research was conducted in the absence of any commercial or financial relationships that could be construed as a potential conflict of interest.
