# Corrigendum: Endoplasmic Reticulum Associated Aminopeptidase 2 (ERAP2) Is Released in the Secretome of Activated MDMs and Reduces *in vitro* HIV-1 Infection

**DOI:** 10.3389/fimmu.2019.02838

**Published:** 2019-12-06

**Authors:** Irma Saulle, Salomè Valentina Ibba, Enrica Torretta, Cecilia Vittori, Claudio Fenizia, Federica Piancone, Davide Minisci, Elisa Maria Lori, Daria Trabattoni, Cecilia Gelfi, Mario Clerici, Mara Biasin

**Affiliations:** ^1^Department of Biomedical and Clinical Sciences L. Sacco, University of Milan, Milan, Italy; ^2^Department of Biomedical Science for Health, University of Milan, Milan, Italy; ^3^Department of Pathophysiology and Transplantation, University of Milan, Milan, Italy; ^4^Don C. Gnocchi Foundation IRCCS, Milan, Italy; ^5^Department of Infectious Disease, ASST Fatebenefratelli Sacco, Milan, Italy; ^6^I.R.C.C.S Orthopaedic Institute Galeazzi, Milan, Italy

**Keywords:** ERAP2, haplotype, HIV-1, MDM, secretion, immune system, CTL, IFNγ

In the published article, there was an error regarding the affiliation for “Cecilia Gelfi.” As well as having affiliation “2”, they should also have “I.R.C.C.S Orthopaedic Institute Galeazzi, Milan, Italy.”

The authors apologize for this error and state that this does not change the scientific conclusions of the article in any way. The original article has been updated.

