# Supplementary material for: Endoplasmic Reticulum Associated Aminopeptidase 2 (ERAP2) Is Released in the Secretome of Activated MDMs and Reduces in vitro HIV-1 Infection
Source: Front Immunol. 2019 Jul 16;10:1648. doi: 10.3389/fimmu.2019.01648 (PMC6646713; doi:10.3389/fimmu.2019.01648)
Supplement: Supplementary file 1 [file Table_1.pdf]

Table S1. ERAP2 (A) and ERAP1 (B) peptides identified by LC-MS/MS analysis, performed on gel bands in the range 105-120 KDa, digested with trypsin.

A

Sequence	Start position	End position	Mass	Missed cleavages	Charges	Score	Posterior Error Probability (PEP)
FDLGSYDIR	887	895	1084.5189	0	2	54.157	0.031258
ILAVTDFEPTQAR	193	205	1459.7671	0	2	78.934	0.00030139
ILYALSTSK	824	832	994.5699	0	2	69.423	0.013583
LIELGMEGK	840	848	988.52632	0	2	82.426	0.003614
LNIPTDVLK	784	792	1011.5964	0	2	80.438	0.011098
TDTLDLPEK	609	617	1030.5183	0	2	72.23	0.010143
TQNLAALLHAIAR	852	864	1390.8045	0	3	26.124	0.040988
YYVAMDFQAK	162	171	1234.5692	0	2	72.547	0.0016477

Protein: ERAP2_HUMAN Endoplasmic reticulum aminopeptidase 2
Accession number: Q6P179
N° of identified peptides: 8 Unique peptides: 8

B

Sequence	Start position	End position	Mass	Missed cleavages	Charges	Score	Posterior Error Probability (PEP)
ASLINNAFQLVSIGK	637	651	1573.883	0	2	59.542	0.000012376
DMNEVETQFK	691	700	1239.544	0	2	41.448	0.02147
EMFDDVSYDK	431	440	1247.502	0	2	45.137	0.014643
ESALLFDAEK	329	338	1121.561	0	2	57.55	0.026351
EYLSADAFK	450	458	1042.497	0	2	62.546	0.021959
HLAISNMPLVK	213	223	1221.69	0	2	78.334	0.00038694
ILASTQFEPTAAR	176	188	1403.741	0	2	78.934	0.00030139
MAFPCFDEPAFK	189	200	1458.631	0	2	57.859	0.0012734
SGIVQYLQK	459	467	1034.576	0	2	60.895	0.023789
SQIEFALCR	799	807	1122.549	0	2	98.299	0.0062316
TQEFPQILTLIGR	829	841	1514.846	0	2	45.359	0.0061572
YQFSLSSTEK	789	798	1188.566	0	2	117.89	0.00061685

Protein: ERAP1_HUMAN Endoplasmic reticulum aminopeptidase 1
Accession number: Q9NZ08
N° of identified peptides: 12 Unique peptides: 12
