# Supplementary figures and images for: Endoplasmic Reticulum Associated Aminopeptidase 2 (ERAP2) Is Released in the Secretome of Activated MDMs and Reduces in vitro HIV-1 Infection
Source: Front Immunol. 2019 Jul 16;10:1648. doi: 10.3389/fimmu.2019.01648 (PMC6646713; doi:10.3389/fimmu.2019.01648)

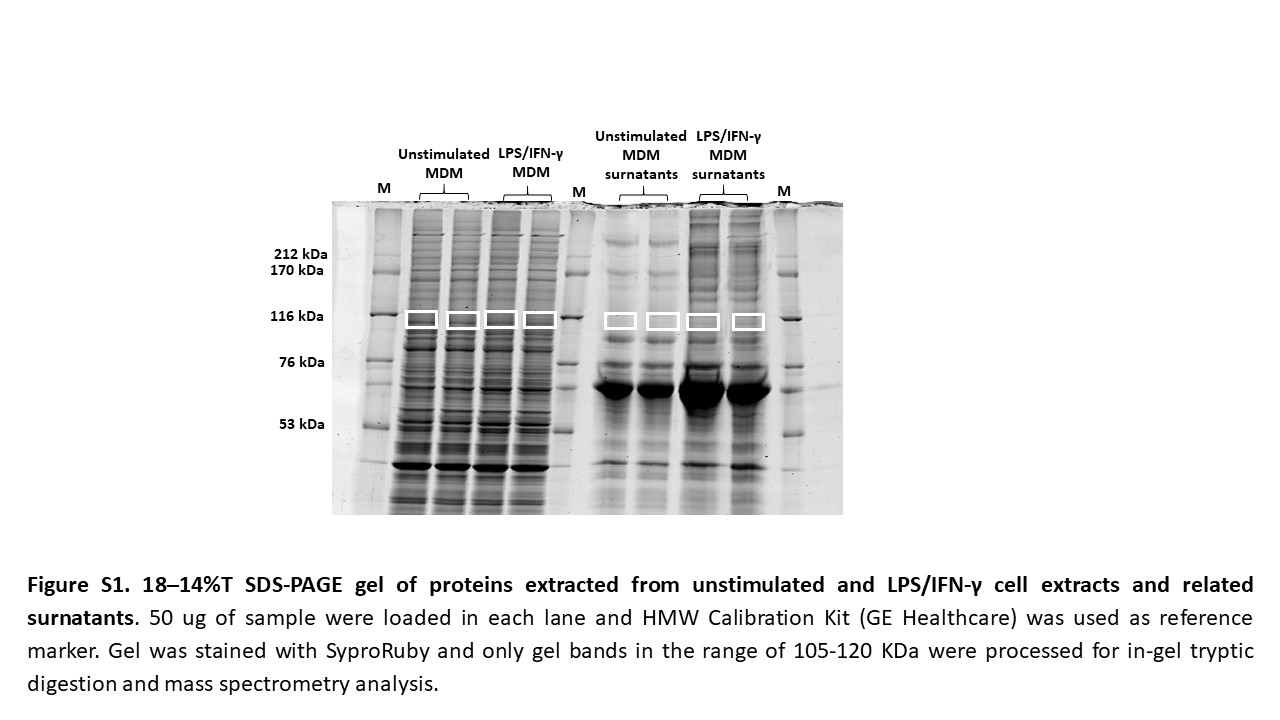

Supplement: Supplementary file 2 [file Image_1.jpg]

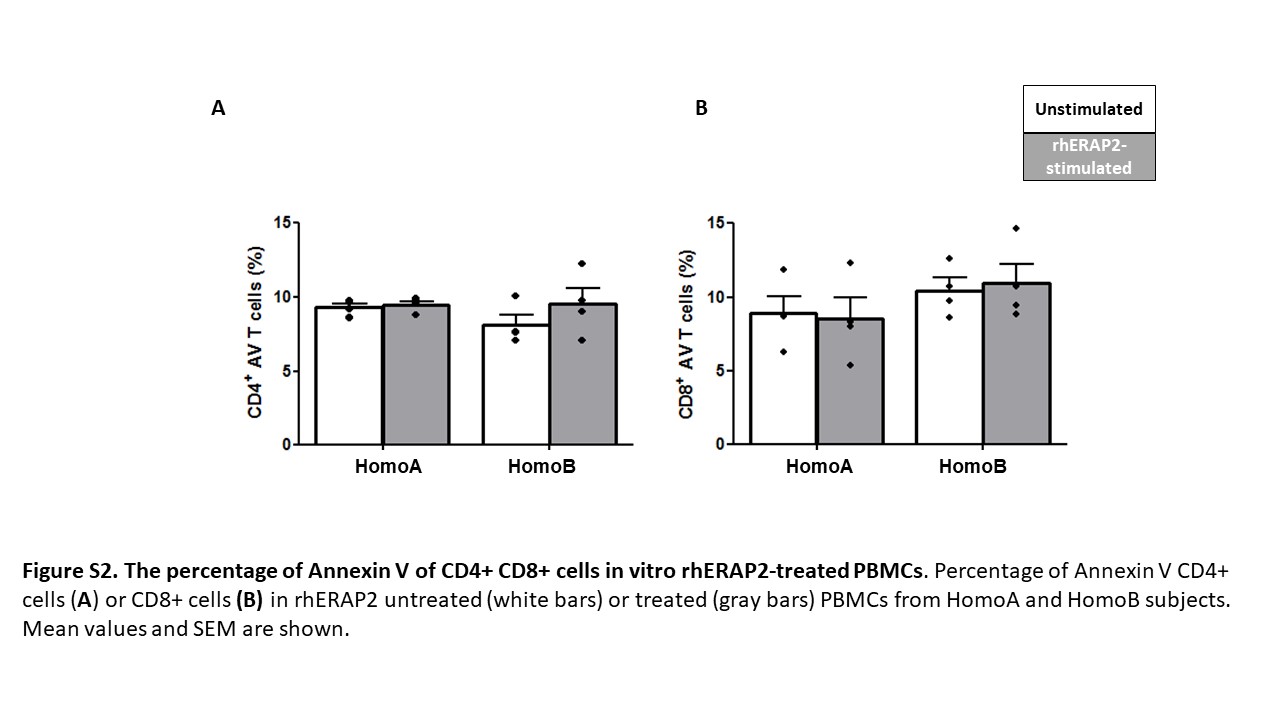

Supplement: Supplementary file 3 [file Image_2.jpg]

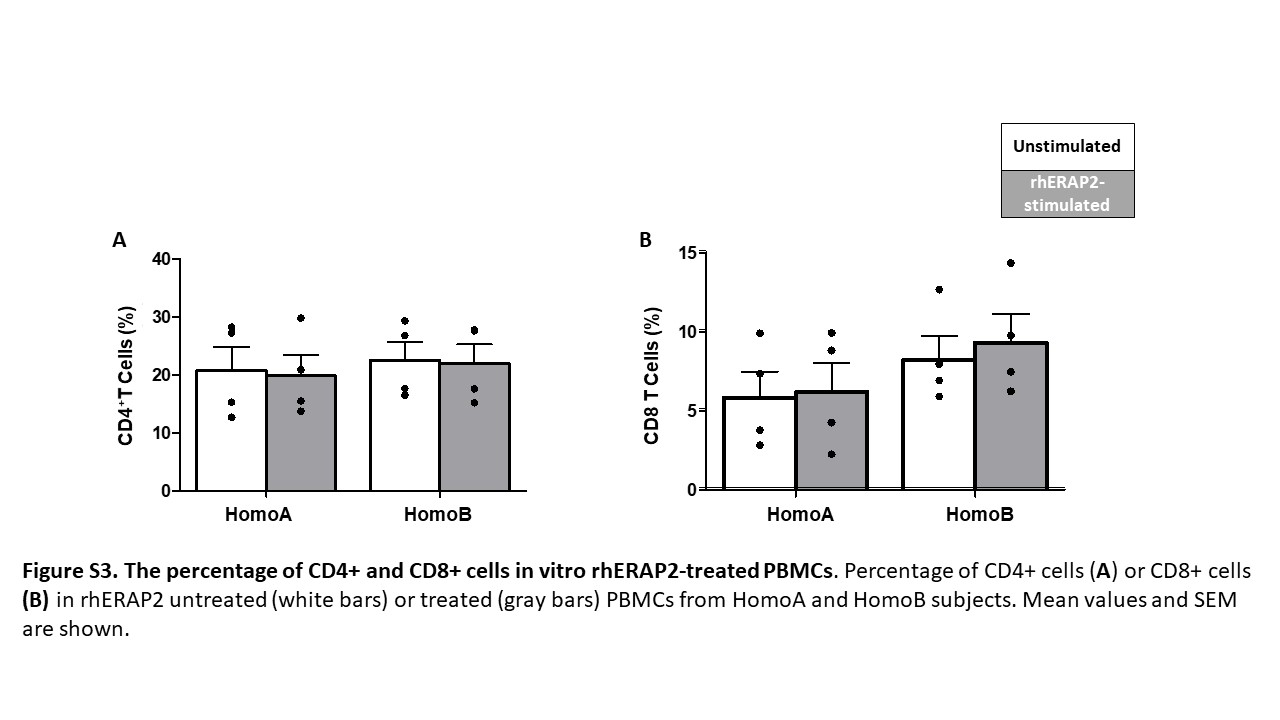

Supplement: Supplementary file 4 [file Image_3.jpg]
